# On the Manufacture and Mounting of Mineral Teeth

**Published:** 1849-01

**Authors:** W. T. G. Morton

**Affiliations:** Surgeon Dentist, Boston.


### ARTICLE II.

### *On the Manufacture and Mounting of Mineral Teeth.* By DR. W. T. G. MORTON, Surgeon Dentist, Boston.

### CHAPTER I.—*Preparations for Impressions.*

Impressions are taken either by means of plaster of paris or gutta percha. I use the latter generally when any teeth are left in the jaw, and when I do not intend to swage up an edge. After being prepared by immersion in hot water, it is placed in a cup of suitable size, and introduced into the mouth, and pressed firmly and perpendicularly against the part to be fitted with the artificial teeth. This pressure should be continued steadily until the jaw is completely imbedded in the gutta percha, which is pressed carefully against the inside and outside of the jaw. Now continuing the perpendicular pressure with the left hand, apply with the right hand a small piece of ice to any part of the gutta percha most convenient, in order to hasten the process of hardening. In a few moments the impression may be removed and immediately immersed in a vessel of ice-water previously prepared for the purpose. Should there be any teeth remaining in the impression which has been taken, fill the impression of the teeth with wax, after which it should present the appearance of an impression of a jaw with the teeth cut off.

*Plaster Models and Annealers made from Gutta Percha Impressions.*

The impression should now be encircled with a strip of paper sufficiently wide to raise the model three-quarters of an inch above the surface of the impression. Then mix the finest plaster of paris with water, until it acquires the consistence of cream, and fill the impression; at the same time gently tap the under surface of the frame, the object of which is to prevent air-bubbles from forming in the plaster model. In order to separate the model thus made from the gutta percha, in most cases a gentle tap only is necessary; but if this does not succeed, heat the plaster until you can separate it, and if filled properly, a model is obtained as smooth as glass, from which the cast is made. In heating the plaster, great care must be taken not to injure the shape of the impression. The impression should now again be encircled with a strip of paper as before, and filled with a paste composed of equal parts of sand and plaster, for the purpose of obtaining an annealer, which is removed in the manner before described. The wax with which the impressions of the teeth were filled is then to be removed, and a piece of steel wire an inch in length placed in the impression of each tooth. The impression is then filled as before, and again suffered to harden, after which it should be immersed in warm water to the depth of the impression, which will immediately render the gutta percha soft enough to be removed without injury to the teeth. We have now a model with the teeth on, which, with the first, should be varnished.

*Plaster Impressions, and Models made from Plaster of Paris Impressions.*

Impressions are taken with plaster of paris as follows: For impressions for a complete set, (in which case only they are taken in this way,) a cup of the proper size is first selected. A sufficient quantity of plaster is reduced to a paste and placed in the cup, which is then introduced into the mouth and pressed against the jaw until it is sufficiently hard to be removed without breaking. It is then removed, and suffered to remain till it is more perfectly hardened. The process of preparing the impressions is as follows: trace the muscles of the jaws upon the impressions, and then pare them down to the mark traced. It is then oiled, and a strip of paper placed round it, as in the case of the gutta percha, before described. The impression is then filled with plaster prepared as before. After this has been permitted to harden, it is taken in the hand and rapped until the two parts separate. We then, of course, have an exact model of the jaw, which should be varnished. The impression is then to be filled with equal parts of sand and plaster, as before described, which is also suffered to harden.

### *Air Chamber.*

Upon the portion of the model forming the roof of the mouth, a piece of wax is placed, of a triangular form, and of the thickness and about the circumference of a cent, varying in size in proportion to that of the jaw.

### *Zinc Casts.*

We next proceed to obtain a cast, which is done by making an impression of the model in fine moulding sand. This should then be encircled with an iron ring. Pure zinc is then melted and poured into the impression. The cast thus obtained is then removed from the ring, and imbedded in sand, with the jaw uppermost; then a ring is placed over it to receive the lead of which the female part should be made.

### *Fitting the Plates.*

These two parts are then separated, and a pattern of sheet-lead is cut of the size and shape which the plate is intended to cover. The plate being procured, take a pair of ball-plyers, and bend the plate somewhat to the shape of the jaw, and after annealing, swage gentle between the male and female cast. Between each swaging, anneal the plate. The plate is then tried upon the model, which should fit so accurately that it may be held under water without moisture penetrating between the plate and the model. It often happens that the plate cannot be made to lie down in all parts after it is swaged up. In this case it is to be laid upon the annealer, and bound down with binding wire until it fits every part accurately. Then anneal. Sometimes the wire cannot be brought to bear upon all parts of the plate. This may be remedied by placing thick pieces of copper under the wire before annealing.* The plate may then be placed in the mouth, to which it should adhere with sufficient strength to resist the process of mastication without being displaced. The same process, with the exception of the air-chamber, is used in fitting the plate to the lower jaw.

### *Articulating Models.*

The plates having been ascertained to be correct, the next step is to make an articulating model. This is done as follows: Place upon the plate soft wax where it is supposed that the teeth are to stand. Then place the plates in the mouth, and direct the patient to sit erect, in order that he may close his mouth naturally. If the models do not then articulate correctly, they should be altered until they do, having regard to the exact length, prominence and thickness of the teeth. These steps having been accurately taken, the patient should close his mouth naturally, while the operator runs a pin from one model to the other, and marks the centre of the mouth upon the wax. Then placing a finger nail of each hand gently between the lower jaw and the plate, he should direct the patient to open his mouth carefully, lest he should disturb the articulations. The upper plate is then loosened with the right hand, and the whole carefully removed from the mouth. If a part of a set only is

* The same method of procedure may be adopted after the teeth have been upon the plate.

to be inserted, a similar method is to be used. Particular care should be used, in so shaping the wax as to have the teeth bear the hardest upon the inner edges, and to have the double teeth of the upper jaw run across those of the lower, the object of this being to drive the upper plate home to its place in the act of mastication. This being done, the model is separated, leaving the plate and the wax upon each jaw, which now exactly represents the teeth.

### *Models for Making the Teeth.*

The shrinking that is allowed in making the teeth, which is about one-eighth, should now be added in red wax, to that portion of the wax which represents the antagonizing surface of each model. The plaster model is now varnished, and the wax model oiled. The cavity of the model of each jaw is then filled with the paste of plaster of Paris, which should not only fill the cavity entirely, but should also extend to the outer edge of the wax. The mark representing the centre of the jaws is then transferred to the plaster models: The teeth are then marked off upon the wax, according to their proper division. The mark between the eye-teeth and the bicuspides on each side is also transferred to the plaster models. The wax is then removed, and a heavy coat of varnish applied to the parts not previously varnished. The model is now ready for making the teeth.

[To be continued ]

